# Groundwater Flow Processes and Human Impact along the Arid US-Mexican Border, Evidenced by Environmental Tracers: The Case of Tecate, Baja California

**DOI:** 10.3390/ijerph15050887

**Published:** 2018-04-30

**Authors:** Jürgen Mahlknecht, Luis Walter Daessle, Maria Vicenta Esteller, Juan Antonio Torres-Martinez, Abrahan Mora

**Affiliations:** 1 Escuela de Ingeniería y Ciencias, Tecnológico de Monterrey, Av. Eugenio Garza Sada Sur No. 2501, Monterrey CP 64849, Nuevo León, Mexico; ja.torres@itesm.mx (J.A.T.-M.); abrahanmora@itesm.mx (A.M.); 2 Instituto de Investigaciones Oceanológicas, Universidad Autónoma de Baja California, Carretera Transpeninsular Tijuana-Ensenada No. 3917, Fraccionamiento Playitas, Ensenada CP 22860, Baja California, Mexico; walter@uabc.edu.mx; 3 Centro Interamericano de Recursos del Agua (CIRA), Facultad de Ingeniería, Universidad Autónoma del Estado de México, Cerro de Coatepec, Ciudad Universitaria, Toluca CP 50110, Estado de México, Mexico; mvestellera@uaemex.mx

**Keywords:** groundwater geochemistry, environmental isotopes, groundwater contamination, arid climate, Tecate, Baja California, Mexico

## Abstract

With the increasing population, urbanization and industry in the arid area of Tecate, there is a concomitant increase in contaminants being introduced into the Tecate River and its aquifer. This contamination is damaging the usable groundwater supply and making local residents and commercial enterprises increasingly dependent on imported water from the Colorado River basin. In this study we apply a suite of chemical and isotopic tracers in order to evaluate groundwater flow and assess contamination trends. Groundwater recharge occurs through mountain-block and mountain-front recharge at higher elevations of the ranges. Groundwater from the unconfined, alluvial aquifer indicates recent recharge and little evolution. The increase in salinity along the flow path is due to interaction with weathering rock-forming silicate minerals and anthropogenic sources such as urban wastewater, residual solids and agricultural runoff from fertilizers, livestock manure and/or septic tanks and latrines. A spatial analysis shows local differences and the impact of the infiltration of imported waters from the Colorado River basin. The general trend of impaired water quality has scarcely been documented in the last decades, but it is expected to continue. Since the groundwater system is highly vulnerable, it is necessary to protect groundwater sources.

## 1. Introduction

**Figure 1:**
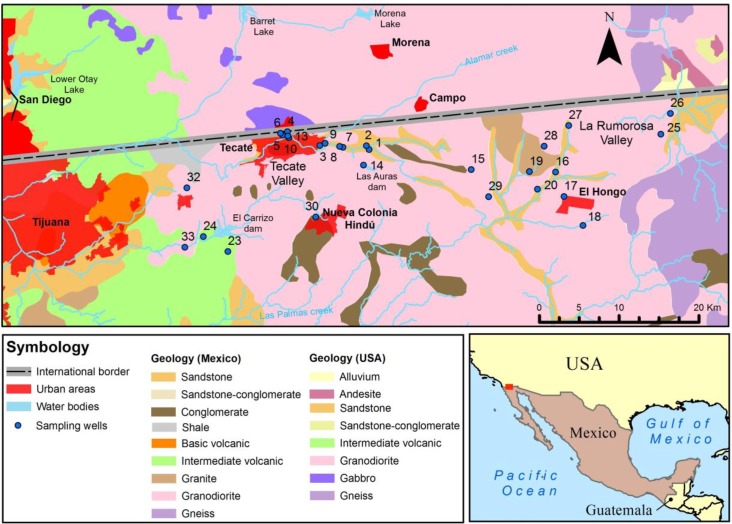
Map of the study area showing the location of the sampled wells.

The peninsula of Baja California in northwestern Mexico has few available water resources and is one of the most arid areas in Mexico. The Tecate River is a sub-basin of the Tijuana River basin, a coastal watershed in Baja California adjacent to the USA-Mexico international border that discharges into the Pacific Ocean (Figure 1). In 2000, the groundwater resources provided about thirty percent of Tecate’s potable water supply [1], decreasing to only twenty percent in 2015 [2]. In addition, with the increase in population, urbanization, and industry in Tecate, there is a concomitant increase in contaminants being introduced into the Tecate River and groundwater resources. The Tecate River, which under natural conditions is an ephemeral stream, has become a perennial one with poor water quality downstream of the discharges. Major point sources of pollution to the river include the discharge of poorly treated sewage from the Tecate municipal plant, the discharge of waters containing high organic matter from a brewery, and effluent discharge from a manufacturing plant complex which includes a large metal-working industry and the effluent from a slaughterhouse [3,4]. Further, it is thought that the aquifer is being degraded by leaking septic tanks, underground storage containers holding fuel products and other chemicals, and agricultural run-off [5].

This contamination is reducing the usable groundwater supply, making local residents and commercial enterprises more dependent on imported water from the Colorado River basin through the 130-km Rio Colorado-Tijuana Aqueduct [2,6]. The volume of water imported from the Colorado basin is susceptible to international agreements, depending on water availability in the USA, and also to climate change factors [7]. The annual extraction of groundwater amounts to ca. 12 million m^3^, of which 35.7% is utilized for public-urban use, 23.5% for agricultural use, 4.3% for livestock use, 16.9% for industrial use, 0.9% for services, and 18.7% for multiple uses [2].

Previous studies in the Tecate aquifer agree that, in general, groundwater was of good quality in terms of salinity (TDS = 300–1200 mg L^−1^). The water type has been described as mixed with a tendency towards bicarbonates [8]. Available information on groundwater in two wells ca. 50 km East from Tecate indicates that TDS and sulfate concentrations were around 1200 mg L^−1^ and 450 mg L^−1^, respectively, with a total hardness of up to 716 mg L^−1^, which could be associated with the local presence of limestone deposits and a limestone quarry [9].

The Tecate area has a rugged landscape with small intermountain valleys where groundwater storage is small in size [10]. Relatively little information is available about its hydrogeological settings, well construction, and water table configuration. Thus, in the present paper we develop a cost-effective method for evaluating groundwater quality and dynamics, which may help water managers in decision making and create higher awareness among local stakeholders about local water resources and their management.

Mathematical tools based on hydraulic data are an excellent way to understand groundwater dynamics and chemical transport. They are however normally time-consuming and expensive. Thus, we propose an alternative using environmental tracers. Chemical constituents in groundwater are not only useful to characterize the chemistry and to evaluate the water quality, but also to understand local trends, to identify hydrochemical processes and to predict the evolution of groundwater chemistry. Isotope ratios help to study the origin and transformation processes in groundwater. Stable isotopes of the water molecule, deuterium (^2^H) and oxigen-18 (^18^O), serve for hydrological fingerprinting.

Radioactive isotopes are useful to evaluate groundwater residence times and eventually to find the relation between these and groundwater vulnerability. Tritium (^3^H) is an environmental pulse tracer derived from the atmosphere that is used as a dating tool for young water, i.e., for timescales of 50 years or less [11,12]. Dating with ^3^H is based on knowledge of the local input function and the measured concentration of ^3^H in the groundwater. The ^3^H concentration in the atmosphere—and thus in young groundwater—recently decreased in North America from its maximum value at the bomb-tritium peak of the 1960s, ultimately reaching pre-bomb values around 1992 [13].

Radiocarbon (^14^C) is the leading tool for estimating the age of paleo groundwater and fossil groundwater, which can be used to date samples that are hundreds of years to approximately 35,000 years old [14,15]. Radiocarbon dating is usually performed by measuring the loss of the parent radionuclide in the dissolved inorganic carbon of a given groundwater sample. Because chemical reactions and the evolution of carbonate systems may dilute the initial ^14^C activity in the dissolved inorganic carbon (DIC), the age must be adjusted geochemically [14,16,17]. Carbon-13-mixing models are very useful in correcting ^14^C ages. These adjustment models use measured carbon-13 (^13^C), combined with ^14^C activity and a series of other parameters (e.g., alkalinity, pH, and temperature) [18,19].

In the present study these tools are applied all together as a suite [20,21], in order to evaluate groundwater flow patterns, groundwater residence times and to assess contamination trends and aquifer vulnerability to surface sources.

## 2. Study Area

Overall, rainfall in Tecate is scarce and cyclonic, occurring during the winter season from December to March, while the dry season is from May to September (a cold semi-arid climate, according to Köppen). The average yearly temperature for the Tecate catchment is 16.5 °C, with a gradient from 15.0 (east) to 18.0 °C (west). The average annual precipitation and evapotranspiration (Turc formula) are 321 mm and 317 mm, respectively [8,22].

Groundwater recharge to the Tecate aquifer is mainly driven by local rainfall and runoff from the Laguna Mountains (USA) and the Sierra Juarez (Mexico) which form part of the Peninsular Range. An estimate of the annual water balance for this aquifer by [8] suggests 10.1 Mm^3^ natural plus induced recharge and 11.8 Mm^3^ discharge, resulting in a water deficit of −1.7 Mm^3^. The mountain-front type of groundwater recharge is typical for valley aquifers in mountainous watersheds in arid and semi-arid regions [23,24].

The rocks of the area are grouped into two hydrogeological units, constituting of a permeable and an impervious unit [8] (Figure 1). The first one consists of granular fragments of fluvial and alluvial origin. The fluvial deposits consist of gravels, sands and clays in smaller quantities, located mainly along the streambeds. The lower, impermeable unit is composed of metamorphic and intrusive igneous rocks—such as granite and diorite—that cover most of the area [8]. These igneous rocks are altered and fractured by weathering processes, representing the development of a secondary permeability. This unit serves as a lateral boundary for the aquifer and transmits the precipitation to the permeable unit, but its water storage is insignificant.

The aquifer system comprises metamorphic and igneous intrusive rocks (granodiorite, tonalite and diorite), overlain by discordant outcrops of continental clastic rocks (alluvial sediments) [9,25,26]. The aquifer is unconfined and has an approximate thickness of 12–18 m [10], with a moderate to high permeability.

Granodiorite contains principally quartz, plagioclase, and biotite. Tonalite contains quartz SiO, andesine (Na,Ca)(Si,Al)_4_O_8_, and small amounts of orthoclase (KAlSi_3_O_8_). Diorite contains 2/3 plagioclase (Na,Ca)(Si,Al)_3_O_8_ and 1/3 mafic minerals (hornblende Ca_2_(Mg,Fe,Al)5(Al,Si)_8_O_22_(OH)_2_, biotite K(Mg,Fe)_3_AlSi_3_O_10_(OH,F)_2_, pyroxene (Ca,Mg,Fe,Mn,Na,Li)(Al,Mg,Fe,Mn,Cr,Sc,Ti)(Si,Al)_2_O_6_). Chemically, this indicates a relatively homogenous aquifer system with predominantly silicate weathering [2]. 

## 3. Materials and Methods

### 3.1. Field Work and Laboratory

Twenty-six wells were sampled in December 2014 for major and minor chemical constituents (Na^+^, K^+^, Ca^2+^, Mg^2+^, ${\mathrm{HCO}}_{3}^{-}$, Cl^−^, ${\mathrm{SO}}_{4}^{2-}$, ${\mathrm{NO}}_{3}^{-}$, F^−^, Sr^2+^, Si, Fe), stable isotope ratios (^2^H, ^18^O, ^13^C in DIC), as well as isotopic activity (^3^H, ^14^C in DIC). Physicochemical parameters were measured in situ and recorded after the variables had stabilized. A pre-calibrated portable multi-meter (Orion Star A329, Thermo Fisher Scientific, Waltham, MA, USA) was used for electrical conductivity (EC), temperature (T), pH and dissolved oxygen (DO). Alkalinity was determined in the field by titration with 0.02 N H_2_SO_4_. Water samples were collected and stored in HDPE bottles at 4 °C and then sent to the laboratory for analysis within a week. Aliquots for cations were filtered through 0.45 µm cellulose acetate membrane and acidified through addition of high purity nitric acid (pH ≤ 2). Dissolved cations and anions were measured using inductively coupled plasma mass spectrometry (ICP-MS) and ion chromatography (IC), respectively, following standard methods [27].

Samples analyzed for their δ^18^O and δ^2^H ratios were collected in 100 mL glass bottles and measured with an isotope ratio mass spectrometer (MM 903; VG Isogas Ltd., Middlewich, UK), using the H_2_O-H_2_ equilibration method (^2^H), which has an analytical precision of ±1.0‰, and the H_2_O-CO_2_ equilibration method (^18^O), which has an analytical precision of ±0.15‰. These measurements were performed at the Environmental Isotope Laboratory of the Department of Geosciences, University of Arizona. The results were reported as δ-values with respect to Vienna Standard Mean Ocean Water (VSMOW).

Tritium, ^14^C-DIC and ^13^C-DIC were measured in 19 out of 26 samples. Tritium was analyzed at the Environmental Isotope Laboratory, University of Arizona (Tucson, AZ, USA). The electrolytical enrichment method used was similar to that of Östlund and Werner [28]. Counting was performed using a Quantulus 1220 liquid scintillation spectrophotometer (PerkinElmer, Waltham, MA, USA). Tritium concentrations are reported in tritium units (TU), in which 1 TU is equal to one tritium atom in 10^18^ hydrogen atoms. The detection limit was 0.3 TU and the analytical precision was typically better than ±10%. Radiocarbon activity was measured by accelerator mass spectrometry at the NSF-Arizona Accelerator Facility for Radioisotope Analysis (Tucson, AZ, USA) and reported as percent modern carbon (pMC). The analytical precision (1-σ) ranged from 0.1 pMC (for pMC values near zero) to 0.5 pMC (for pMC values near 100 pMC). Carbon-13 measurements were performed using a Finnigan Delta S spectrometer (Thermo Fisher Scientific, Waltham, MA, USA) at Environmental Isotope Laboratory, University of Arizona (Tucson, AZ, USA), on carbon dioxide previously released by acidifying sample water [29]. These results are reported in δ^13^C_DIC_ with respect to the Vienna Pee Dee Belemnite (VPDB) standard and with an analytical precision of 0.15‰ (1-σ).

### 3.2. Interpretation

Samples with a chemical charge balance error >10% were discarded for interpretation. The significant difference of mean values between this sampling campaign and a sampling campaign in 1981, was statistically evaluated by the *t*-test. The null hypothesis adopted in the *t*-test was that concentration levels of a particular solute were not significantly different between the years. This hypothesis was rejected when calculated *p*-values were less than 0.05.

The water classification was done with a Hierarchical Cluster Analysis (HCA) scheme, considering 18 variables measured in all samples (T, pH, EC, OD, Na^+^, K^+^, Ca^2+^, Mg^2+^, ${\mathrm{HCO}}_{3}^{-}$, Cl^−^, ${\mathrm{SO}}_{4}^{2-}$, ${\mathrm{NO}}_{3}^{-}$, F^−^, Sr^2+^, Si, Fe, ^2^H, ^18^O). Iteratively neighboring points (samples) using Ward’s linkage rule were linked through a similarity matrix [30]. The squared Euclidian distance was selected as the similarity measurement. For the cluster analysis, log-normal distributed data were previously log-transformed, and all of the variables standardized (z scores).

Saturation indexes were calculated in order to elucidate the equilibrium state of water with respect to minerals. The saturation index (SI) is defined as SI = log (IAP/K) where IAP is the ion activity product and K is the equilibrium constant. If SI = 0, the solution is in equilibrium with the mineral phase and no reaction is expected. If SI > 0, groundwater is oversaturated with respect to the particular mineral, which means that the mineral phase may precipitate to achieve equilibrium. Finally, if SI < 0, groundwater is undersaturated with the mineral phase, which means that dissolution is required to reach equilibrium. The PHREEQC program [31] was used for the calculation of saturation indexes.

Unadjusted ^14^C ages were calculated from measured ^14^C activities in DIC by using the Libby half-life and assuming initial ^14^C activity of 100% modern carbon (pMC). Age adjustments were made using the Fontes & Garnier and Pearson model [18,32]. This model considers a two-stage evolution of recharge waters, accounting for the dissolution and isotopic exchange of carbonate minerals with CO_2_ in the unsaturated zone, as well as isotopic exchange with carbonate rocks in the saturated zone [14,21,33,34]. The ^14^C activity and δ^13^C value of dissolved carbonate minerals were set as 0 pMC and 0‰ VPDB, respectively. The ^14^C activity of soil CO_2_ in all samples is set to 102 pMC, as it appears to be the ^14^C activity in samples from recharge areas [2]. The δ^13^C value of soil CO_2_ was set according to predominant natural vegetation in recharge areas. Tecate’s vegetation includes coastal sage scrub, chaparral, pine forest, oak forest, and desert scrub. Also important are meadowlands, aquatic and riparian areas [35]. Thus, the δ^13^C value is set as −24‰ VPDB to account for the mainly C3 plants in the recharge area. Isotopic fractionation factors were calculated using the methods of Vogel et al. [36]; Mook et al. [37]; and Deines et al. [38].

## 4. Results

### 4.1. Chemical Characterization

Table 1 presents the results for the physicochemical parameters, the chemical concentrations, stable isotope ratios and radioactive isotope activities of the groundwaters studied. The pH range is 7.0–8.6 (average 7.6 ± 0.3), the temperature is 13.6–21.8 °C (average 18.4 ± 2.1 °C) and EC is 437–7480 µS cm^−1^ (average 1427 ± 1326 µS). Dissolved oxygen varies between 2.8 and 13.1 mg L^−1^ (average 6.8 mg ± 2.5 mg L^−1^), indicating oxic conditions in groundwater. The dominance of cations is Na^+^ > Ca^2+^ > Mg^2+^ >> K^+^ and of anions is ${\mathrm{HCO}}_{3}^{-}$ > Cl^−^ > ${\mathrm{SO}}_{4}^{2-}$ > ${\mathrm{NO}}_{3}^{-}$ >> F^−^. The concentrations of Cl^−^, ${\mathrm{NO}}_{3}^{-}$, ${\mathrm{SO}}_{4}^{2-}$ and F^−^ range from 34 to 1584 mg L^−1^, 0.2 to 35.5 mg L^−1^, 12.6 to 574.8 mg L^−1^ and 0.3 to 2.2 mg L^−1^, respectively. The higher values are found to the west (Tecate Valley) and to the extreme east (Rumorosa Valley) of the study area (Figure 1).

Seventy-seven percent of the samples have a TDS >500 mg L^−1^, and 31% have TDS >1000 mg L^−1^. This means that every third sample can be classified as slightly saline water. Gangster et al. reports an increasing deterioration in water quality starting in 1992 [35]. From previous studies for the study area, only one reports groundwater water chemical parameters (INEGI, 1982). This study considers a field sampling campaign in winter 1981, with 16 sites that match this study. Thus, it was possible to compare mean values between 1981 and 2014.

Table 2 summarizes the average values of physicochemical parameters for groundwater from 1981 and 2014. As shown, groundwater samples from 2014 had higher Ca^2+^, Mg^2+^, Na^+^, K^+^, Cl^−^, ${\mathrm{HCO}}_{3}^{-}$, and ${\mathrm{NO}}_{3}^{-}$ concentrations, and hence higher EC values, than groundwater samples from 1981. The concentrations for ${\mathrm{SO}}_{4}^{2-}$ remained practically unchanged. The hydrochemical difference was confirmed by a *t*-test (Table 2); among the physicochemical parameters measured, only Ca^2+^ and ${\mathrm{HCO}}_{3}^{-}$ were significantly different (*p* value < 0.05) between the two sampling campaigns, while the rest did not show any significant difference. From this is can be concluded that there exists an increasing trend between 1981 and 2014, however, it is not significant for most physicochemical parameters.

With ~52% of equivalent anion chemistry, ${\mathrm{HCO}}_{3}^{-}$ is the dominant species among anions. Weathering of alumino-silicate rocks and the contribution of CO_2_ gas dissolution are supposed to be the primary sources of ${\mathrm{HCO}}_{3}^{-}$. Biodegradation of organic matter and root respiration increases CO_2_ pressure in soils. The infiltrating water absorbs CO_2_ by generating HCO_3_. Chloride makes up roughly 31% of anion chemistry. Generally, Cl^−^ has its origin in atmospheric precipitation or mixing with seawater. The Na/Cl molar ratio ranges from 0.7 to 3.2 (average 1.7). Halite dissolution would produce a Na/Cl ratio equal to 1.0, and seawater close to 0.85. Na/Cl ratios decrease with increasing salinity, which is an indication that the evaporation effect is not a dominant process. Concentration mainly by evaporation process would leave the Na/Cl ratio unchanged. Rather, the higher Na/Cl ratio suggests silicate weathering and/or anthropogenic sources such as agriculture runoff or waste as the main processes. Sulfate concentration is in the order of 16% of total anion chemistry. Lower concentrations in the study area were most probably due to rock weathering, while higher concentrations above 60 mg L^−1^ were attributable to the urban area indicating anthropogenic sources like industrial effluents. The significant correlation between SO_4_^2−^ and Cl^−^ concentrations (r^2^ = 0.79; t = 0.000) confirms this assumption. Finally, ${\mathrm{NO}}_{3}^{-}$ concentrations in groundwater constituted ~2% of total anionic concentrations. Nitrate generally derives from agricultural fertilizers, human and animal excreta, biological fixation and nitrification processes, and atmospheric precipitation [39,40,41]. Fluoride concentration in groundwater (up to 2.2 mg L^−1^) exceeds the national drinking water standard (1.5 mg L^−1^) in only one sample. In volcanic environments, F^−^ usually originates from weathering of fluoride-bearing rock forming minerals like fluorite, biotite and muscovite [39,42].

Alkali metals Na^+^ (43–748 mg L^−1^) and K^+^ (1.3–186 mg L^−1^) account together for about 48% of the total cations equivalency, respectively. The origin of these ions in the studied waters is primarily weathering of rock-forming silicates such as plagioclase, andesine and orthoclase, which are part of a granodiorite, diorite and tonalite rock matrix. Alkaline earth metals Ca^2+^ (28.1–374.3 mg L^−1^) and Mg^2+^ (12.2–247.7 mg L^−1^) account for 31% and 21% of total cations, respectively. The presence of these ions may indicate weathering of minerals such hornblende, olivine, pyroxene, plagioclase and, to a smaller extent, calcite dissolution. Iron (<0.18 mg L^−1^) is detected in only 27% of samples (detection limit = 0.02 mg L^−1^). This suggests association of Ca^2+^ and Mg^2+^ with plagioclases rather than with mafic minerals.

### 4.2. Hydrochemical Clusters

**Figure 2:**
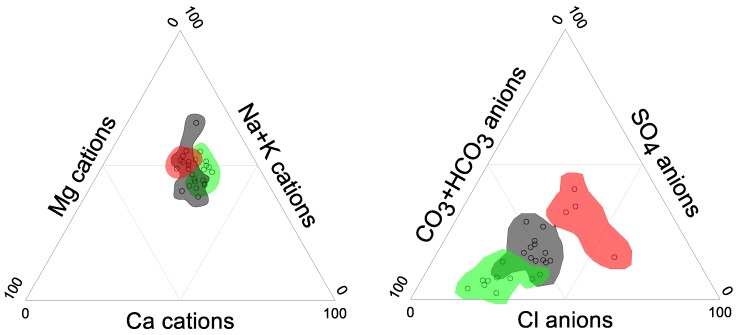
Trilinear diagrams for cations and anions indicating the HCA statistical groups: Group 1, in green, stands for samples located in the triangle of Tecate-La Rumorosa-El Hongo. Group 2, in grey, represent samples in Tecate, La Rumorosa and El Hongo. Group 3, in red, considers samples south and southeast of Tecate.

In order to understand local chemical trends, it is necessary to classify samples according to their chemical similarity and geographical correspondence. A hierarchical cluster analysis classifies samples into 3 groups: Group 1 is located inside the triangle with vertices Tecate, La Rumorosa and El Hongo; Group 2 is located in Tecate City and the towns of La Rumorosa and El Hongo; and Group 3 is located towards the El Carrizo dam and Nueva Colonia Hindú locality south and southwest of Tecate City (Figure 1). Trilinear diagrams for cations and anions show the chemical differences between the three groups (Figure 2).

While the differences in the cation concentrations are relatively small, for the anions there is a clear increasing trend of relative Cl^−^ and ${\mathrm{SO}}_{4}^{2-}$ content from Group 1 to Group 3, while lowering the relative ${\mathrm{HCO}}_{3}^{-}$ contribution. Group 1 is a mixed-HCO_3_ to Na-HCO_3_ water type and Group 2 is a mixed-HCO_3_ to Na-HCO_3_ to mixed-mixed type. There is slight overlapping between Group 1 and 2. Finally, Group 3 is a mixed-mixed to Na-mixed type. This sequence of groups displays a relatively uniform geochemical evolution suggesting water-rock interaction, i.e., weathering of mostly acid silicate rocks among other processes.

### 4.3. Statistical Variation of Selected Variables

**Figure 3:**
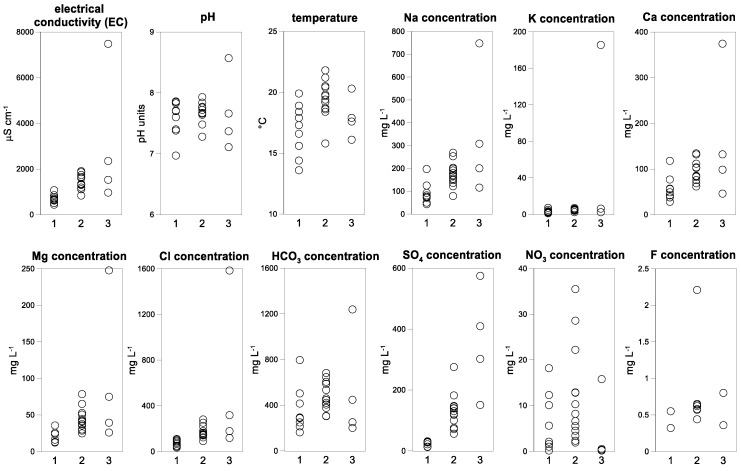
Ranges of selected variables, according to the 3 groups.

The classification of samples into groundwater groups also reveals a more detailed picture about groundwater evolution, when looking to the comparison of statistics of selected variables in form of boxplots (Figure 3). Group 1 has a lower mineralization (average 695 µS cm^−1^) and temperature (17.0 °C) in comparison to the other groups, reflecting a low circulation and recent recharge and pristine conditions in the hilly central to northeastern area. Group 2, located mainly in Tecate Valley shows a general trend of increased temperature (19.6 °C) and mineralization (1424 µS cm^−1^), suggesting more evolved groundwater. The concentration of ${\mathrm{SO}}_{4}^{2-}$ (131.0 mg L^−1^) and ${\mathrm{NO}}_{3}^{-}$ (11.9 mg L^−1^) is almost five times and two times higher than the first group, respectively, which indicates a significant human impact on groundwater resources. The distribution of nitrate concentrations shows that high values are found in both urban (Tecate city) and rural (around El Hongo) areas. The elevated concentrations in urban Tecate point to infiltration of treated wastewater from the Tecate river and leaching from waste disposal. In the rural area, the high concentrations indicate agricultural activities involving nitrogen components such as fertilizers or organic byproducts, septic tanks and livestock manure near El Hongo. Finally, Group 3 integrates samples located in the southwestern study area with a temperature of 18.0 °C and largely variable concentrations, which is generally higher and more heterogeneous than previous groups. Similarly, this group shows an important deterioration in water quality regarding Cl^−^, ${\mathrm{NO}}_{3}^{-}$ and ${\mathrm{SO}}_{4}^{2-}$ concentrations.

### 4.4. Identification of Hydrogeochemical Processes

**Figure 4:**
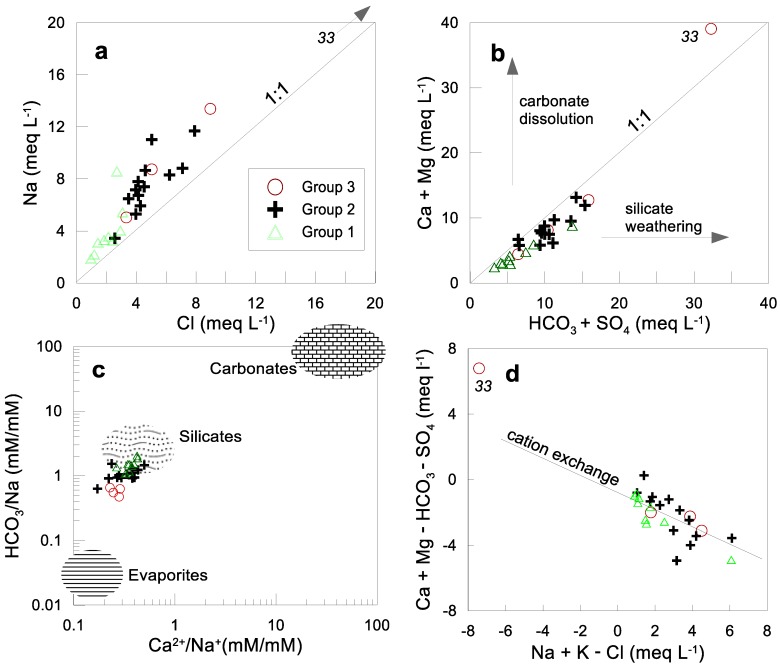
Scatterplots used to assess hydrogeochemical processes: (**a**) Na vs. Cl; (**b**) (HCO_3_ + SO_4_) vs. (Ca + Mg); (**c**) Ca/Na vs. HCO_3_/Na; (**d**) Na + K - Cl vs. Ca + Mg - HCO_3_ - SO_4_.

Scatter diagrams show a strong correlation between Cl^−^ and Na^+^, and Na^+^ excess in relation to Cl^−^ (Figure 4a). This reflects that besides halite dissolution, other processes such as plagioclase weathering (mainly albite) and/or Na-Ca ion exchange could be taking place. Another alternative would be the effect of sodium-rich wastewater disposal from households [43]. This could explain why the Na/Cl ratio is higher in urban Group 2 and 3 than in rural Group 1. The plot (Ca + Mg) versus (HCO_3_ + SO_4_) in Figure 4b shows that all points fall below the 1:1 equiline, which suggests that the Ca and Mg chemistry is largely explained by silicate weathering processes. In order to evaluate this assumption, a plot of Ca/Na vs. HCO_3_/Na is given (Figure 4c). It appears from this molar relationship that groundwater samples are all close to typical values for silicate weathering tending to evaporate weathering. Therefore, other processes such as carbonate dissolution are of little importance for groundwater acquiring ion concentrations during water-rock interaction. Ion exchange processes can be evaluated using the relationship between (Ca + Mg - HCO_3_ - SO_4_) and (Na + K - Cl). If ion exchange plays a predominant role in the system, then the values should fall along a line with a slope of −1. This is not the case for our study area (Figure 4d). The data show that the average slope is −0.80 (r^2^ = 0.84), which suggests that cation exchange of Ca and Mg for Na is important, but other processes such as albite weathering are also present. The slopes of each group are relatively similar to each other and range from −0.72 to −0.83.

**Figure 5:**
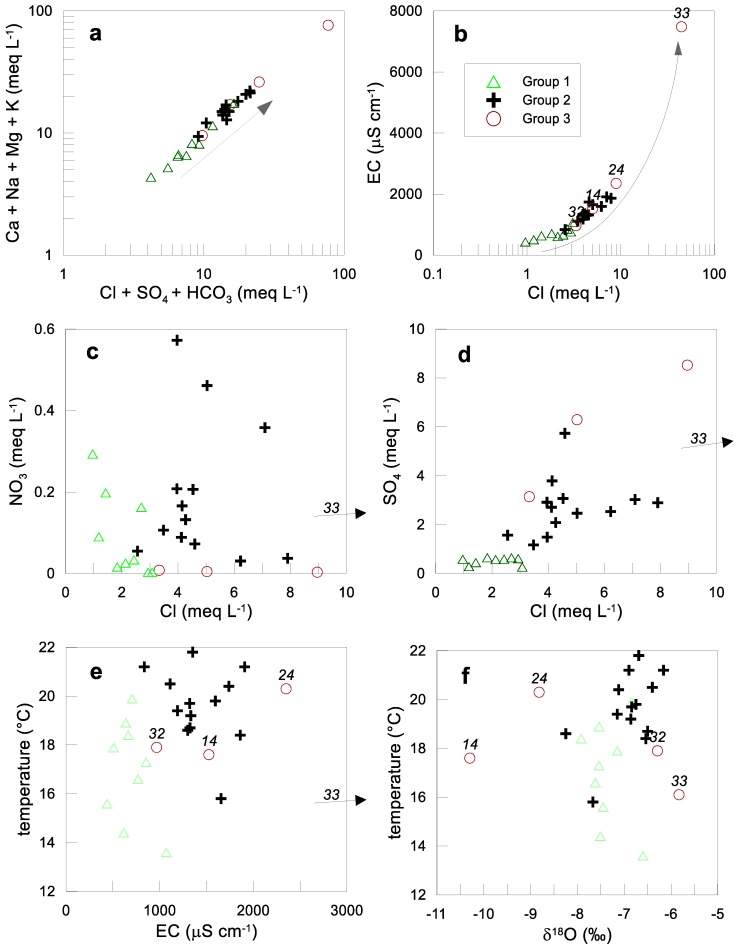
Scatter plots for evaluation of hydrogeochemical processes: (**a**) (Ca + Na + Mg + K) vs. (Cl + SO_4_ + HCO_3_); (**b**) EC vs. Cl; (**c**) NO_3_ vs. Cl; (**d**) SO_4_ vs. Cl; (**e**) salinity vs. temperature; (**f**) oxygen-18 vs. temperature.

Water infiltrated recently has low concentrations of chemical constituents. As water moves along the flow path and the travel time is longer, more ions are acquired along the flow path. In our case, Group 1 is the least mineralized water and Groups 2 and 3 show increased TDS (Figure 5a). EC and Cl^−^ concentrations increase exponentially along with the flow (Figure 5b). Group 1 water is associated with recent recharge and short transit times (local flow), while group 2 and 3 with greater distances and longer residence times. Group 1 also shows lower temperatures, Cl^−^ and ${\mathrm{SO}}_{4}^{2-}$ concentrations compared to Group 2 (Figure 5b,d). Group 2 exhibits the largest variation and highest values of ${\mathrm{NO}}_{3}^{-}$ among all groups. Group 3 displays the highest ${\mathrm{SO}}_{4}^{2-}$ values but the lowest ${\mathrm{NO}}_{3}^{-}$ concentrations (Figure 5c,d). In general, salinity increases with temperature, i.e., a trend from a low temperature/salinity in Group 1 to elevated temperature/salinity in Group 3 (Figure 5e). Given the fact that the depth of the water table ranges from 2.1 to 15.3 m [2], the aquifer system is prone to local recharge even of poor water quality. 

### 4.5. Geochemical Trends

**Figure 6:**
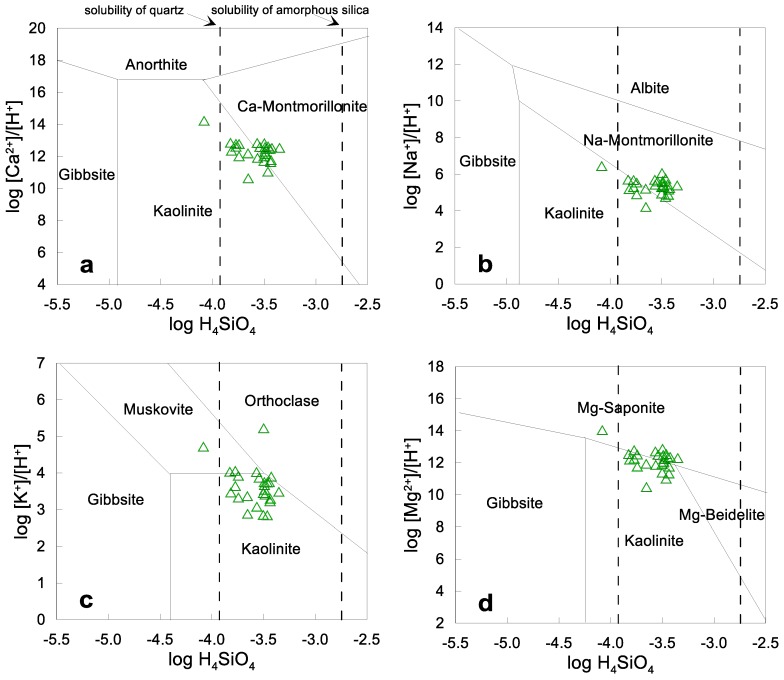
Stability of (**a**) Ca-Al silicate phases; (**b**) Na-Al silicate phases; (**c**) K-Al silicate phases; and (**d**) Mg-Al phases in aqueous solution at 25 °C and 1 atm pressure with groundwater data of the study area.

Stability diagrams of Ca-Al, Na-Al, Mg-Al, and K-Al silicate phases are useful to study the occurrence of incongruent dissolution (e.g., [41,44]). Figure 6a shows that most water samples are found in the limit between the stability field of kaolinite and Ca-Montmorillonite, and some samples are located in the Ca-montmorillonite, Na-montmorillonite and Mg-smectite fields, respectively. Thus, albite, anorthite, gibbsite, muscovite and orthoclase (microcline) would be unstable, facilitating their dissolution and contribution to the presence of Na^+^, Ca^2+^ and Mg^2+^ in groundwater. Based on these data, the reactions involving the incongruent dissolution of albite to kaolinite or Na-montmorillonite, and anorthite to kaolinite or Ca-montmorillonite, and orthoclase/muscovite to kaolinite would be mostly favored in this aquifer. This means that infiltrating water enriched with soil CO_2_ reacts with silicate minerals from the host rocks, albite, anorthite and orthoclase, leaching out Na^+^, K^+^, Mg^2+^ and ${\mathrm{HCO}}_{3}^{-}$, and converting in kaolinite, montmorillonite and silica. Besides, other processes occur such as ion exchange on clayey surfaces that set free Na^+^.

**Figure 7:**
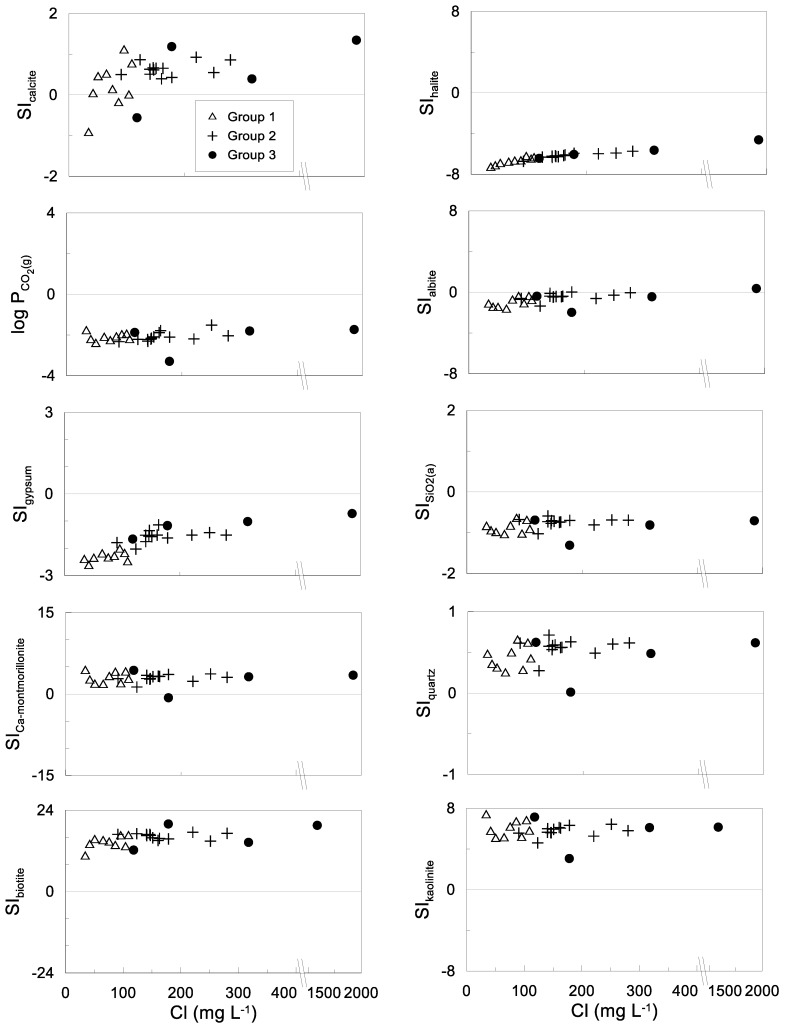
Saturation indexes versus chloride concentrations of relevant reacting mineral phases.

Saturation indexes are valuable in predicting the presence and trend of reactive minerals in groundwater. The results show that the samples of all groups are under-saturated with respect to halite, and silica, signaling their dissolution from mineral phases (Figure 7). There is a trend of water towards saturation with respect to albite, meaning that this mineral tends to dissolve in water under the given conditions. The calcite saturation index shows a trend from under-saturation to over-saturation. The large variation of SIs for calcite is remarkable. The over-saturation is probably due to the dissolution of Ca-containing minerals such as anorthite. The figures for Ca-montmorillonite, quartz, biotite and kaolinite exhibit over-saturation, which suggests a trend to precipitation of these mineral phases. This is congruent with the most common minerals that compose the rocks in the study area and with stability diagrams (Figure 6). The P_CO2_ values (10^−3.3^–10^−1.5^) are in the order of soils where bacterial oxidation of vegetation and respiration of CO_2_ in the root zone occurs. 

### 4.6. Stable Isotopes

**Figure 8:**
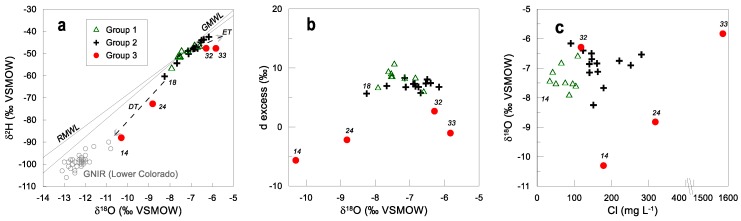
(**a**) δ^18^O and δ^2^H in groundwater from the study area; (**b**) δ^18^O and ^2^H excess; (**c**) δ^18^O compared to chloride concentration. Labels indicate selected sample numbers. ET means evaporation trend. DT means dilution trend.

Stable water isotopes are a powerful means to study the water cycle. Specifically, the origin and exchange processes may be studied using isotope ratios and deuterium excess. ^18^O and ^2^H ratios vary from −10.3 to −6.2‰ and from −88.0 to −42.5‰, respectively (Table 1). A comparison of the isotope ratios in groundwater from the study site with those of the global meteoric water line [45] and the regional meteoric water line (RMWL) (International Atomic Energy Agency-Global Network of Isotopes in Precipitation (IAEA-GNIP) station for Chihuahua), suggests that groundwater sampled in this study is of meteoric origin and has undergone varying degrees of evaporation (Figure 8a). Most samples are located below the present-day RMWL and define a linear trend described by the relationship δ^2^H = 4.25δ^18^O − 18.71 (evaporation trend, ET), which is indicative of evaporation during recharge. The evaporation trend has an intercept on the meteoric water line at −7.65‰, close to the composition of modern precipitation in this area, indicating that this groundwater is derived from modern precipitation that is exposed to evaporation during its passage through the soil and unsaturated zone to reach the water table. The range of values from δ^2^H and δ^18^O is similar to those reported in the areas of La Mision and Valle de Guadalupe [46,47] which are near (ca. 100 km) to the study area and adjacent to the Pacific Ocean.

Another subset of samples shows a trend to more depleted values in comparison to RMWL (No. 14, 24, 18, 25). Sample No. 14 is close to the Las Auras dam, and sample No. 24 is close to the El Carrizo dam. This is indicative that these waters are modified due to a mixture of water from surface water bodies which are supplied by water with different meteoric origin, such as that derived from the Colorado River-Tijuana aqueduct that supplies these dams. A comparison with synoptic data retrieved from the Global Network of Isotopes for Rivers (GNIR-IAEA) for the lower Colorado River basin evidences this mixture of water (DT; Figure 8a), being sample No. 14 closest to the isotopic composition of river water.

Deuterium excess varies mostly between 5‰ and 10‰ (Figure 8b). Low deuterium excess values (<10‰) may be caused by post-condensation evaporative effects, whereby falling rain drops in warm regions having low relative humidity undergo slight re-evaporation before hitting the ground, a feature generally seen only in summertime [48,49]. From these observations, it can be concluded that there are at least two recharge mechanisms: (i) recharge from mountainous areas, which enters and travels through the fractured/porous aquifer as horizontal flow; (ii) vertical recharge to the aquifer as a product of rainwater or anthropogenic activities at the surface. Not only are Cl^−^ concentrations and chemistry quite different between Groups 1 and 2, but also their isotopic signature. Most points of Group 1 have −7.5‰ as δ^18^O, whereas most points of Group 2 have −6.5/−7‰ (Figure 8c). In general, the stable water isotopes are not strongly affected by evaporation as expected from the dry climate of the region. This can be explained by the rapid infiltration process in the mountainous recharge areas via fractures. With regard to Group 1, the δ^18^O signature increases with cumulative salinity, and for Groups 2 and 3, no clear trend is observable (Figure 5e). This is in part due to the effect of water from the aqueduct, at least in several samples.

### 4.7. Groundwater Age

Groundwater age represents a direct indicator of the transit time of water through a catchment and is therefore a useful parameter for describing catchment processes and movement of contaminants. The results for ^3^H and ^14^C are presented in Table 1. Tritium activities range from <0.5 to 7.4 TU. The IAEA-GNIP station for Chihuahua (~1100 km SE from Tecate) reports that the activity of ^3^H has been decreasing since the 1960s and its current level is about 2 to 3 TU [21]. Radiocarbon values vary from 90.6 to 108.8 pMC. 

**Figure 9:**
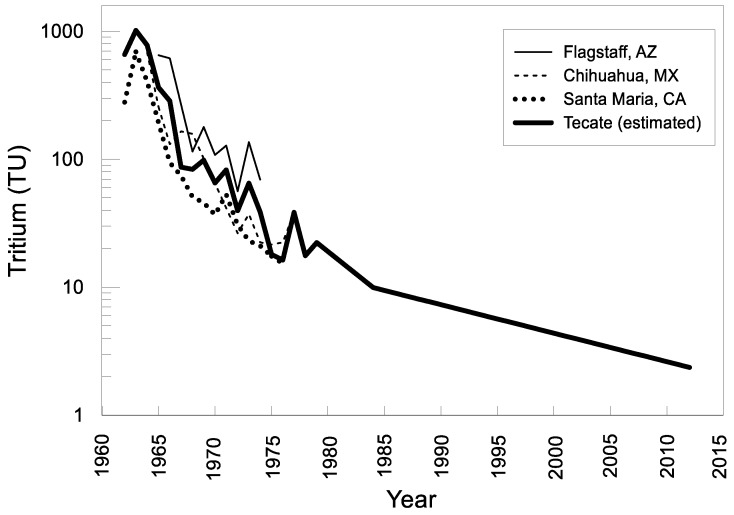
Estimated input function of tritium using the IAEA-GNIP network data of the three closest stations Santa María (California), Flagstaff (Arizona), and Chihuahua (Mexico).

The local input function of ^3^H was compiled based on the three closest stations of the IAEA-GNIP -in Flagstaff, Arizona (35°7′48′′ N and 111°40′12′′ W), Santa Maria, California (34°53′0′′ N and 12°27′0′′ W) and Chihuahua, Mexico (28°37′48′′ N and 106°4′12′′ W) by taking the inverse-distance-squared weighted interpolation of the annual average input values of the three stations (Figure 9). The sampling records for Flagstaff, Santa Maria and Chihuahua cover 1961 to 1976, 1961 to 1976 and 1962 to 1988, respectively. For input values after 1988, an annual decrease of the tritium activity of 5.5% was calculated [14,21]. This is consistent with data from Eastoe [50], who reports an average concentration of 5 TU in rainwater for the Tucson basin from 1992–1998, and data from Harris [51] who reports for the Safford basin, Arizona, ^3^H levels during the winter of 1994–1995 of 2–3 TU and surface water containing 8–10 TU, which reflect the remaining effects of the atomic bomb tritium spike. The current level in rainwater is estimated to be in the order of 2 to 3 TU.

**Figure 10:**
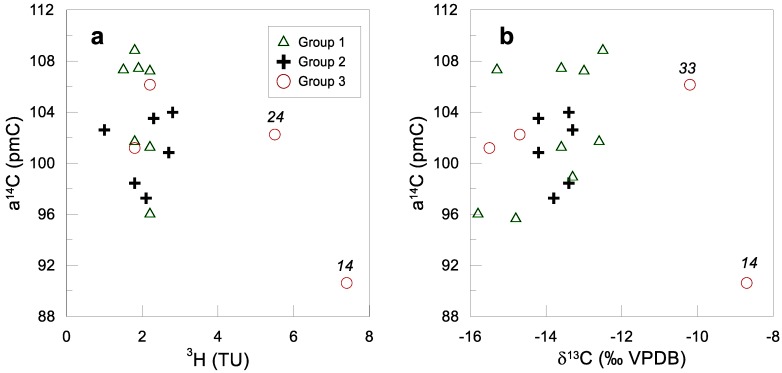
Isotope relationships: (**a**) ^14^C vs. ^3^H; (**b**) ^14^C vs. 13C.

The relation between ^14^C and ^3^H shows two different trends (Figure 10a). Most samples in the study site show a narrow range of ^3^H activity (1.8–2.2 TU), suggesting that recharge from recent years is significant. The exceptions are samples No. 14 and 24. These two samples have relatively high ^3^H activities (5.5 and 7.4 TU, respectively), and are related to groundwater mixing with surface water from the Las Auras and Carrizo dams, as mentioned earlier. In general, the high ^14^C values (>90 pMC) confirm recent recharge and little or no mixture with other older waters. Samples with the highest ^14^C values, in the order of 108 pMC, suggest a recirculation of water in Tecate Valley and consequently a major contribution of bomb-influenced water from the 1950s and 1960s. Carbon-13 ratios vary from −15.4 to −8.7‰. There is a general trend of increasing δ^13^C ratio with increasing ^14^C values, with the exception of sample No. 14, which is more enriched (Figure 10b). If the carbon-containing rocks were an important source for chemical reactions, the relation would be inverse. Also, the general distance between δ^13^C values in groundwater and limestone indicate none or minimal contribution of carbonate rocks. Sample No. 25 and 26 are located in La Rumorosa Valley where marmolized Paleozoic limestone is mined in open quarries. However, the mentioned water samples apparently indicate no influence from carbonate-bearing rocks.

For radiocarbon age dating two different approaches were used, that gave similar results. According to both, the Pearson model and Fontes and Garnier model, all waters are modern (<1000 years old). In general, any estimation is rather uncertain because the age is sensitive to initial values of soil δ^13^C and a^14^C. However, testing these models with changed soil δ^13^C CO_2_ values of ±2‰ results does not change the results. Similarly, a change of initial ^14^C activity for ±3 pMC produces the same result. Still, unless there is field data available for soil ^13^C, it is impossible to estimate more accurate groundwater ages. Independently, it can be stated that practically all samples are modern water, and there is no evidence of admixture of fossil groundwater.

With tritium it was possible to perform a semi-quantitative dating approach. This means that groundwater is modern (post-1952), when ^3^H is larger than the detection limit and ^14^C is higher than 99 pMC; it is pre-modern when ^3^H is not detected and ^14^C is below 99 pMC; and it is mixed when ^3^H is detected and ^14^C is below 99 pMC. In accordance with this preliminary classification, thirteen samples (50%) were modern, two (8%) were pre-modern, and four (15%) represent a mix of pre-modern and post-modern, while the rest (seven samples; 27%) were not measured.

## 5. Discussion

The joint interpretation of hydrogeological, chemical and isotopic data shows that local groundwater recharge for Tecate Valley is derived from local rainfall and mountain-front infiltration. Groundwater in this unconfined aquifer flows in a western direction at a depth of 5 to 30 m below ground. It is a mixed-HCO_3_ to Na-HCO_3_ water type with ^3^H and ^14^C concentrations of 1.0–2.8 TU and 97.3–104 pMC, respectively. It is characterized by relatively high mineralization (1424 µS cm^−1^), nitrate (1.9–35.5 mg L^−1^) and sulfate (56–275 mg L^−1^) contents. These data indicate that infiltration from urban area have deteriorated water quality to a degree that it is not potable or close to not drinkable in 76% of the samples due to the high mineralization. Groundwater from southwest of the Tecate Valley shows admixture of water from the El Carrizo and Las Auras dams. These waters derive from the Río Colorado-Tijuana aqueduct, which are discharged on a riverbed 6 km away from the El Carrizo dam. Like water from the Colorado River with δ^18^O = −12.6 and δ^2^H = −102.4 [52] they have significantly more depleted isotope compositions, and high Cl^−^ and ${\mathrm{SO}}_{4}^{2-}$ concentrations, but very low ${\mathrm{NO}}_{3}^{-}$ [52,53]. The ^3^H values in these samples indicate post-bomb waters. Finally, groundwater from the La Rumorosa Valley at the eastern portion of the study area—a mixed-HCO_3_ to Na-HCO_3_ type—is shallow water, but it has a lower mineralization than Tecate Valley, as well as lower ${\mathrm{SO}}_{4}^{2-}\;$ and Cl^−^ concentrations, but similar high ${\mathrm{NO}}_{3}^{-}$ contents. Groundwater from this area is 5–15 m below ground. Samples from this aquifer are chemically and isotopically similar to those from Tecate Valley. Overall, 64% of the analyzed samples (mainly the waters of Groups 2 and 3) have nitrate concentrations higher than the threshold value (3 mg/L) for anthropogenic influence [40], which suggests nitrate contamination due to infiltration of sewage water, septic deposits, etc. Similarly, 40% of the water samples showed nitrate levels above the Mexican standard (10 mg/L as NO_3_) for drinking water [54], indicating that groundwater should be pretreated for nitrate removal before it can be supplied to population.

The presence of ^3^H in groundwater evidences that recharge has occurred post-1950. Thus, the groundwater is considered modern. Carbon-14 does match in all samples with this trend. Sample No. 32, for example, reflects initial conditions according to hydrogeological settings. Its P_CO2_ is 10^−1.9^ which is similar to soil [14]. This sample has a low alkalinity (${\mathrm{HCO}}_{3}^{-}$ = 201 mg L^−1^), and a trend of calcite dissolution (SI_calcite_ = −0.6) occurring under open conditions (Table 1, Figure 6). The δ^13^C value of this sample (−15.5‰) indicates that the δ^13^Csoil_CO2_ is approximately −25‰ under the given pH and temperature conditions (7.1 pH units and 18 °C). This is congruent with the initial estimate for C3 plants (from -20 to −30‰) considering a vegetation of coastal sage scrub, chaparral, pine forest, oak forest, and desert scrub. 

The travel time of groundwater in wells and springs is indicative of its vulnerability to pollution. The different geochemical ages eventually can be used to evaluate chemical differences in groundwater [20]. In our case, all samples are modern and thus vulnerable, however, no correlation between ${\mathrm{NO}}_{3}^{-}$ or other chemical components concentration with the age of the samples was seen. Thus, it is suggested that the differences in contaminant concentrations are a question of geographic location rather than age differences.

## 6. Conclusions

Human processes associated with urbanization, industry and agriculture have resulted in deterioration in the surface and groundwater quality in the arid Tecate area during the last several decades. Contamination has reduced the usable groundwater supply and made local residents and commercial enterprises more dependent on imported water from the Colorado River. In this study, groundwater flow processes have been investigated using chemical and isotopic evidence. Natural and anthropogenic sources of groundwater contamination have been inspected in addition to hydrogeochemical characterization in relation to groundwater flow. 

Groundwater recharge occurs through mountain-block and mountain-front recharge at higher elevations of the ranges around Sierra Juarez and the Laguna Mountains and local rainfall. Groundwater of the unconfined, alluvial aquifer system is of Na- to mixed-HCO_3_ type and indicates recent infiltration with little evolution and no mixture with hydrothermal fluids. As groundwater moves rapidly from the hills to the valleys, it acquires salinity, specifically, bicarbonate, sodium, chloride, calcium, sulfate, and magnesium and nitrate, in order of importance. The origin of bicarbonate, sodium, calcium and magnesium ions is primarily incongruent weathering of rock-forming silicate minerals.

There are also human impacts on groundwater chemistry that show spatial differences. Groundwater from the central and northeastern rural area have the lowest mineralization and temperatures, predominant water-rock interactions and nearly pristine conditions. Groundwater from urban Tecate and Rumorosa valleys have higher mineralization, with nitrate originating from urban wastewaters, residual solids and agricultural runoff from fertilizers, livestock manure and/or septic tanks in rural areas. Finally, groundwater from the southwestern portion of the study area are affected by mixtures with surface water from the lower Colorado river, with deterioration in water quality with regard to chloride and sulfate.

The general trend of a decrease in water quality has until now, scarcely been documented for the last three decades. It is expected that this trend will continue as industrial, and agricultural activites increase and populations grow according to the “business as usual” model. The groundwater system consisting of small unconfined units are highly vulnerable and groundwater sources need to be protected. Additional studies are necessary to define priority areas for groundwater protection. A water quality monitoring program has to be established in order to elaborate a groundwater management program.

## Figures and Tables

**Table 1:** Physicochemical parameters, chemical concentrations, charge balance error, stable isotope ratios and isotopic activities.

Well ID	Temp	pH	EC	OD	Na	K	Ca	Mg	HCO_3_	Cl	SO_4_	NO_3_	F	Sr	Fe	Si	Charge Balance Error	^18^O	^2^H	^3^H	^13^C	^14^C
(-)	(°C)	(std. Unit)	(µS cm^−1^)	(mg L^−1^)	(mg L^−1^)	(mg L^−1^)	(mg L^−1^)	(mg L^−1^)	(mg L^−1^)	(mg L^−1^)	(mg L^−1^)	(mg L^−1^)	(mg L^−1^)	(mg L^−1^)	(mg L^−1^)	(mg L^−1^)	(%)	(‰)	(‰)	(TU)	(‰)	(pMC)
1	19.9	7.7	706	5.0	76.1	2.5	56.1	23.8	414.3	65.1	31.3	1.0	<0.5	0.264	0.010	9.2	−6.8	−6.84	−46.47	2.2	−13.0	107.2
2	20.5	7.9	1111	7.2	149.0	6.0	61.6	37.4	604.8	123.5	56.2	6.6	0.7	0.422	0.010	10.2	−6.8	−6.40	−43.75	---	---	---
3	18.7	7.8	1323	6.7	179.2	5.7	83.9	40.6	444.7	146.1	130.4	5.5	0.6	0.550	0.010	17.3	4.7	−6.50	−43.94	2.8	−13.4	104.0
4	21.2	7.8	835	7.3	79.0	6.1	68.7	28.5	305.7	90.7	75.4	3.4	<0.5	0.278	0.010	22.7	1.0	−6.16	−42.51	---	---	---
5	20.4	7.5	1738	5.5	199.1	5.3	131.8	65.1	587.9	163.0	275.3	4.5	<0.5	0.761	0.010	19.5	1.9	−7.12	−50.20	---	---	---
6	21.8	7.7	1351	3.8	154.9	5.4	102.5	44.2	376.4	146.9	182.1	10.3	<0.5	0.524	0.010	21.6	5.2	−6.69	−47.75	2.7	−14.2	100.8
7	18.4	7.8	1860	6.6	268.6	4.4	103.7	52.7	646.9	280.3	139.2	2.3	<0.5	0.626	0.010	20.7	−0.5	−6.54	−44.94	1.8	−13.4	98.4
8	19.8	7.8	1594	5.3	190.9	7.0	111.5	50.4	533.1	220.8	121.8	1.9	<0.5	0.418	0.010	16.3	1.7	−6.75	−47.20	2.1	−13.8	97.3
9	21.2	7.3	1907	7.9	202.9	4.8	134.1	78.5	681.9	251.5	145.5	22.2	<0.5	0.841	0.010	22.0	1.6	−6.90	−47.92	---	---	---
10	19.2	7.8	1332	4.3	164.8	4.4	85.1	41.9	405.7	140.5	140.1	12.9	0.6	0.589	0.010	19.4	4.8	−6.86	−47.57	---	---	---
13	19.7	7.5	1319	5.3	170.2	4.2	83.0	40.8	459.1	160.6	147.6	12.8	0.6	0.562	0.010	18.9	−1.0	−6.84	−47.80	---	---	---
14	17.6	8.6	1525	13.1	200.8	6.4	98.5	39.4	250.9	178.3	302.2	0.3	0.4	1.262	0.013	5.2	4.9	−10.30	−88.04	7.4	−8.7	90.6
15	15.6	7.0	437	9.2	43.1	3.5	28.1	12.2	162.5	34.0	28.1	18.2	<0.5	0.238	0.010	13.3	−0.3	−7.45	−48.98	2.2	−15.8	96.0
16	13.6	7.9	1070	5.3	125.2	5.1	77.0	25.5	503.1	109.2	12.6	0.2	0.3	0.826	0.065	10.9	−1.2	−6.60	−46.88	1.8	−12.6	101.7
17	19.4	7.7	1189	8.3	121.9	2.6	85.0	30.1	303.4	140.7	71.4	35.5	0.4	0.651	0.010	26.8	6.6	−7.15	−48.88	---	---	---
18	18.6	7.7	1299	10.8	136.5	2.5	102.8	35.5	445.3	151.3	100.2	8.2	0.6	0.682	0.010	19.7	0.6	−8.25	−60.33	2.3	−14.2	103.5
19	17.3	7.8	854	11.8	197.4	7.2	117.8	35.5	795.7	95.6	31.0	10.1	<0.5	0.457	0.010	9.0	2.8	−7.54	−51.83	1.9	−13.6	107.4
20	18.9	7.9	637	10.8	71.3	2.7	48.9	13.0	288.2	50.5	21.6	12.3	<0.5	0.439	0.010	10.2	−1.4	−7.54	−51.35	1.8	−12.5	108.8
24	20.3	7.4	2350	2.8	307.8	2.4	132.1	74.8	447.6	317.6	409.4	0.2	0.8	0.030	0.180	16.3	2.3	−8.82	−72.73	5.5	−14.7	102.2
25	18.4	7.4	664	7.3	74.9	2.9	42.3	12.5	214.0	86.2	28.5	2.1	<0.5	0.051	0.010	22.0	−2.8	−7.92	−56.76	<0.5	−13.3	98.9
26	15.8	7.6	1655	5.2	253.3	4.8	75.7	24.7	421.1	178.5	118.6	28.6	2.2	0.110	0.010	19.4	7.3	−7.67	−54.40	1.0	−13.3	102.6
27	16.6	7.4	767	5.8	92.7	1.3	57.1	16.1	286.6	104.0	29.4	0.2	0.6	0.086	0.019	18.9	−2.1	−7.62	−51.65	<0.9	−14.8	95.7
28	14.4	7.7	616	6.1	81.9	2.0	37.9	12.7	293.0	75.6	27.7	1.6	<0.5	0.058	0.023	13.3	−9.3	−7.51	−51.60	2.2	−13.6	101.3
29	17.9	7.6	508	5.2	50.4	2.3	37.7	13.0	248.9	41.8	13.8	5.6	<0.5	0.022	0.010	10.9	−6.5	−7.15	−49.04	1.5	−15.3	107.3
32	17.9	7.1	967	5.0	115.8	2.4	46.0	25.9	200.5	118.0	150.9	0.5	<0.5	0.039	0.026	20.6	−3.3	−6.29	−47.64	1.8	−15.5	101.2
33	16.1	7.7	7480	5.8	747.6	185.6	374.3	247.7	1238.7	1584.4	574.8	15.8	<0.5	0.002	0.078	18.7	−0.7	−5.83	−47.67	2.2	−10.2	106.1

**Table 2:** Average, standard deviation (±1σ) and *p*-values of various physicochemical parameters for the Tecate aquifer.

**Year**	**Measures**	**CE (µS/cm)**	**pH**	**Ca (mg L^−1^)**	**Mg (mg L^−1^)**	**Na (mg L^−1^)**
1981	Avg. ± St.dev.	1122 ± 739	8.1 ± 0.4	51.3 ± 16.6	40.6 ± 33.2	138.3 ± 139.5
2014	Avg. ± St.dev.	1622 ± 1642	7.6 ± 0.3	103.0 ± 79.4	49.3 ± 56.7	193.0 ± 163.3
	*p*-value	0.001 *	0.001 *	0.021 *	0.584	0.269
		**K (mg L^−1^)**	**Cl (mg L^−1^)**	**HCO_3_ (mg L^−1^)**	**SO_4_ (mg L^−1^)**	**NO_3_ (mg L^−1^)**
1981	Avg. ± St.dev.	4.3 ± 2.1	175.9 ± 184.7	207.6 ± 45.6	159.0 ± 205.7	7.2 ± 6.3
2014	Avg. ± St.dev.	15.2 ± 45.5	232.5 ± 367.1	472.7 ± 261.6	151.5 ± 161.6	8.8 ± 11.1
	*p*-value	0.346	0.577	0.001 *	0.887	0.640

Note: * means significantly different
